# Targeted demethylation at ZNF154 promotor upregulates ZNF154 expression and inhibits the proliferation and migration of Esophageal Squamous Carcinoma cells

**DOI:** 10.1038/s41388-022-02366-y

**Published:** 2022-09-05

**Authors:** Wenting He, Shiyong Lin, Yi Guo, Yuanzhong Wu, Lu-Lu Zhang, Qiling Deng, Zi-Ming Du, Mingbiao Wei, Weijie Zhu, Wan-Juan Chen, Jian-Yong Shao, Guo-Liang Xu

**Affiliations:** 1 Department of Molecular Diagnostics, Sun Yat-Sen University Cancer Center, State Key Laboratory of Oncology in South China, Collaborative Innovation Center for Cancer Medicine, Guangzhou, China; 2 Department of Endoscopy and Laser, Sun Yat-Sen University Cancer Center, State Key Laboratory of Oncology in South China, Collaborative Innovation Center for Cancer Medicine, Guangzhou, China; 3 Department of Endoscopy, Shantou University Cancer Hospital, Shantou, China; 4 Department of Experimental Research, Sun Yat-Sen University Cancer Center, State Key Laboratory of Oncology in South China, Collaborative Innovation Center for Cancer Medicine, Guangzhou, China; 5 Research and Development Department, Anhui Targene Medical Technology Company Ltd, Wuhu, China

**Keywords:** Oesophageal cancer, Targeted therapies, Cancer screening

## Abstract

Zinc finger protein 154 (ZNF154) is hypermethylated at the promoter in many epithelial-derived solid tumors. However, its methylation status and function in esophageal squamous carcinoma (ESCC) are poorly understood. We found that the ZNF154 promoter is hypermethylated in ESCC and portends poor prognosis. In addition, ZNF154 functions as a tumor suppressor gene (TSG) in ESCC, and is downregulated by promoter hypermethylation. We established a targeted demethylation strategy based on CRISPR/dCas9 technology and found that the hypermethylation of ZNF154 promoter repressed ZNF154 induction, which in turn promoted the proliferation and migration of ESCC cells in vitro and in vivo. Finally, high-throughput CUT&Tag analysis, GEPIA software and qPCR were used to revealed the role of ZNF154 as a transcription factor to upregulate the expression of ESCC-associated tumor suppressor genes. Taken together, hypermethylation of the ZNF154 promoter plays an important role in the development of ESCC, and epigenetic editing is a promising tool for inhibiting ESCC cells with aberrant DNA methylation.

## Introduction

Esophageal cancer (EC) is the sixth deadliest malignancy worldwide, and is classified into squamous cell carcinoma and adenocarcinoma [1]. Esophageal squamous cell carcinoma (ESCC) accounts for 90% of all EC cases worldwide, with a higher incidence in China [2]. In addition, the five-year survival rate of ESCC patients at the advanced stage is less than 10% [3, 4]. Currently, EC is treated by surgical resection, radiotherapy and chemotherapy. However, due to the limited efficacy and severe adverse effects, the outcomes of conventional treatments are still unsatisfactory. Targeted therapies such as monoclonal antibodies have improved the prognosis of EC patients when used in combination with conventional therapies [5]. Therefore, it is crucial to identify novel biomarkers of late-stage EC to prolong patient survival.

Epigenetic aberrations, specifically the hypermethylation of tumor suppressor genes, are frequent events in ESCC [6]. Epigenetics is the study of heritable changes in gene function that are not attributed to the alteration in sequence [7]. Epigenetic modifications like DNA methylation and histone modification regulate chromatin structure, and any aberrations can alter the structure of chromosomes, leading to abnormal gene expression and pathological changes, including cancer [8, 9].

DNA methylation usually occurs on CpG island and silences gene expression by inhibiting the binding of transcription factors with promoters or by recruiting methyl-CPG binding proteins to bind with inhibitory histone modifying enzymes [10, 11]. It occurs at the fifth carbon of cytosine base within the CpG dinucleotide via the addition of a methyl group [12]. The methylation process is dynamic and involves demethylation enzymes which erase 5-methylcytosine (5mC) to restore the unmethylated state [13]. Tet (ten eleven translocation) dioxygenase-catalyzed 5mC oxidation promotes DNA demethylation with Tet catalytic domain (Tet-CD) as the smallest functional module [14, 15]. Tet proteins, including Tet1, Tet2 and Tet3, catalyze the DNA demethylation process in a concerted manner. Tet1 catalyzes the oxidation of 5mC to 5-hydroxymethylcytosine (5hmC), which acts as the first step of demethylation [16**–**18].

Zinc finger proteins (ZNFs) constitute the largest family of transcription factors in the human genome, and bind with the target gene promoters via the zinc finger domain [19**–**21]. A previous study showed that the CpG island of the ZNF154 promoter is hypermethylated in 15 epithelial-derived solid tumors [22]. Since ESCC is also an epithelial-derived tumor, we hypothesized that the CpG island of ZNF154 promotor may be hypermethylated in ESCC, and play a role in cancer development. The CRISPR/Cas9 gene editing tool targets the specific gene through a single guide RNA (sgRNA) [23, 24]. Fusion of the nuclease “dead” Cas9 (dCas9) with the Tet1CD can achieve targeted removal of methylated DNA without the shearing action of Cas9 [13]. In this study, we found that the ZNF154 promoter is hypermethylated, and increases the proliferation and migration of ESCC cells. Using the targeted demethylation strategy based on the CRISPR/dCas9 technology, we proved that ZNF154 promoter methylation is a factor in the development of ESCC. Our results show that epigenetic editing can reprogram the aberrant DNA methylation signature of cancer-related genes, and is therefore a promising therapeutic strategy against ESCC and other cancers.

## Results

### ZNF154 promoter is hypermethylated in ESCC and correlates with poor prognosis

**Fig. 1:**
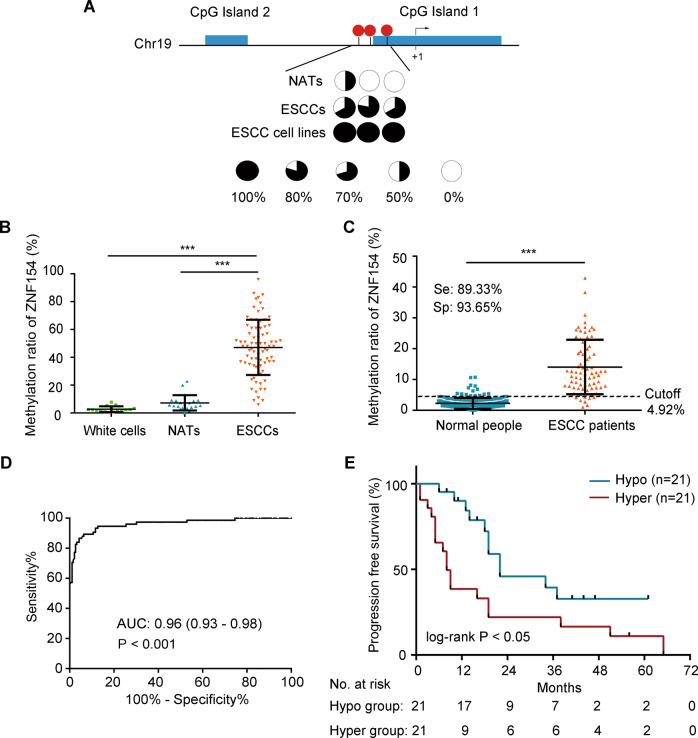
ZNF154 promoter is hypermethylated in ESCC and correlates with poor prognosis. **A** Methylation status of three CpG sites around CpG1 was analyzed by bisulfite sequencing in nine ESCC tissues, four NATs and two ESCC cell lines (Kyse-30 and Kyse-140). The three CpG sites are represented with the red circles. Open and filled circles represent unmethylated and methylated CpG sites respectively. Circles are partially filled according to the percentage of methylation. **B** The methylation ratios of white blood cells from healthy individuals (*n* = 16), NATs (*n* = 21) and ESCC tissues (*n* = 78). **C** The methylation ratios of normal people (*n* = 189) and ESCC patients (*n* = 75) from EECs are shown. **D** Receiver operating characteristic (ROC) curve of the MRs for distinguishing between normal people and ESCC patients. **E** The 5-year PFS of ESCC patients in the hypermethylated group (MR > 48%) and hypomethylated group (MR ≤ 48%). ***P < 0.001 2-tailed *t* test. Equation for MR: methylated droplets (MD)/(MD + unmethylated droplets (UMD)). NAT unpaired normal adjacent tissues, ESCC esophageal squamous carcinoma, MR methylation ratio, Se sensitivity, Sp specificity, Hyper hypermethylation, Hypo hypomethylation.

Two CpG islands (CGIs) around the transcriptional start site (TSS) were identified by using MethPrimer with the default criteria [25] (Fig. 1A). Given that CGIs in the promoter are frequently methylated, we performed bisulfite sequencing to assess CpG methylation status within the two CGIs in nine ESCC tissues, four unpaired normal adjacent tissues (NATs), as well as two ESCC cell lines (Kyse-30 and Kyse-140). The methylation status of three CpG sites around CGI1 were heavily methylated in ESCC cell lines (100% methylation in both cell lines) and tissues (67%, 78% and 67%), while NATs showed relatively lower methylation level (50%, 0% and 0%) (Fig. 1A). To further verify hypermethylation of this region in ESCC patients, we detected the methylation ratio (MR) in 78 ESCC tissues, 21 NATs and 16 healthy human leucocytes by droplet digital PCR (ddPCR). The MR in ESCC tissues (47.13% ± 19.86) was significantly higher than that in the NATs (7.31% ± 5.56, *P* < 0.001) and leucocytes (2.74% ± 2.06, *P* < 0.001) (Fig. 1B, Supplementary Fig. 1). We next analyzed the MR of the ZNF154 promoter in esophageal exfoliated cells (EECs) of 189 normal people and 75 ESCC patients, and detected significantly higher level of methylation (*P* < 0.001) in the ESCC patients (11.99%) versus the healthy controls (1.74%) (Fig. 1C). Furthermore, ROC curve analysis showed high diagnostic efficacy of hypermethylated ZNF154 promoter for ESCC with 89.33% sensitivity and 93.65% specificity, and the optimal cut-off value of 4.92% (Fig. 1D).

To determine the prognostic relevance of ZNF154 promoter hypermethylation, we analyzed the methylation ratios in the tumor tissues from 78 ESCC patients, of which 42 patients had been regularly followed-up. We assessed the 5-year progression-free survival (PFS) rate of these 42 patients that were demarcated into the hyper- and hypomethylated groups based on the median MR of 48%. As shown in Fig. 1E, the 5-year PFS rate of the hypermethylated group was significantly lower compared to that of the hypomethylated group (11% vs 32.79%, *P* < 0.05), indicating a high possibility of relapse among ESCC patients with hypermethylated ZNF154 promoter within the 5-year follow-up period. In addition, ZNF154 promoter hypermethylation was correlated significantly with the advanced T stage (*P* < 0.05) in the cohort of 78 ESCC patients (Table 1). Taken together, the ZNF154 promoter is frequently hypermethylated in ESCC and correlates with poor prognosis.

**Table 1:** Relationship between the MR of ZNF154 promotor and clinicopathologic parameters of 78 ESCC tissues.

		ZNF154 methylation ratio		*P* value
Variables	Number of cases, %	High, % (MR > 48%),	Low, %(MR ≤ 48%),	
Age, years old				
≥61	44 (56.4)	21 (47.7)	23 (52.3)	0.475
<61	34 (43.6)	19 (55.9)	15 (44.1)	
Sex				
Male	65 (83.3)	36 (55.4)	29 (44.6)	0.188
Female	13 (16.7)	4 (30.8)	9 (69.2)	
Pathologic grade				0.865
I	28 (35.9)	14 (50.0)	14 (50.0)	
II–III	50 (64.1)	26 (52.0)	24 (48.0)	
TNM stage				0.249
I–II	38 (48.7)	15 (39.5)	23 (60.5)	
III–IV	40 (51.3)	21 (52.5)	19 (47.5)	
T stage				<0.05
T1–2	21 (26.9)	5 (23.8)	16 (76.2)	
T3–4	57 (73.1)	31 (54.4)	26 (45.6)	
N stage				0.438
N0	34 (43.6)	14 (41.2)	20 (58.8)	
N1–3	44 (56.4)	22 (50)	22 (50)	
Tumor location				0.650
Upper/Middle	54 (69.2)	24 (44.4)	30 (55.6)	
Lower	24 (30.8)	12 (50)	12 (50)	

*MR* methylation ratio, *TNM* tumor-node-metastasis.

*P* values were calculated using χ2 test, or Fisher’s exact test.

### ZNF154 functions as a tumor suppressor in ESCC and is downregulated by promoter hypermethylation

**Fig. 2:**
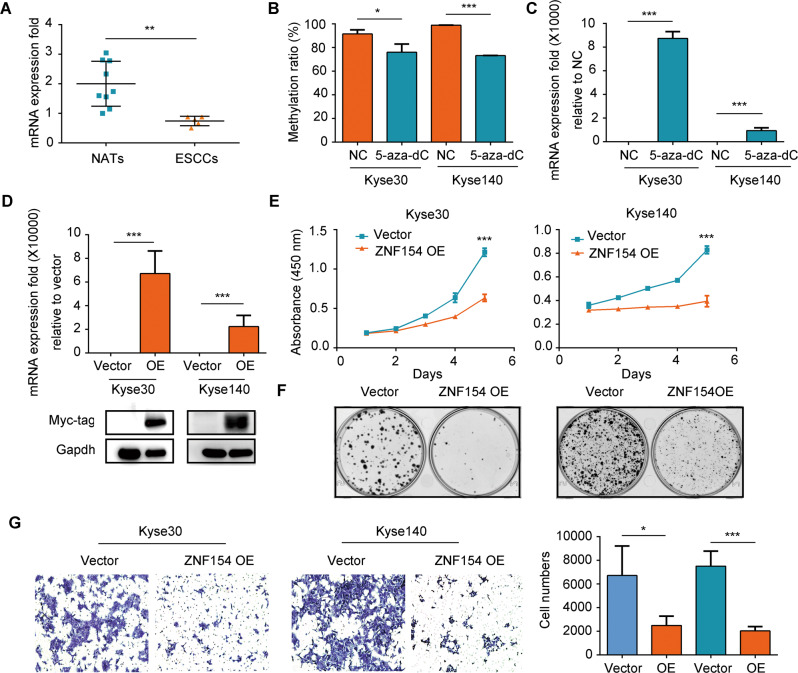
ZNF154 functions as a tumor suppressor gene in ESCC and is downregulated by promoter hypermethylation. **A** ZNF154 mRNA levels in the NATs (*n* = 9) and ESCC tissues (*n* = 5). **B** MR of ZNF154 promoter in ESCC cells treated with 5-Aza-dC (2 µM). **C** ZNF154 mRNA levels in ESCC cells treated with 5-Aza-dC. **D** ZNF154 mRNA and Myc-tag protein levels in Kyse30 and Kyse140 cells with or without ZNF154 overexpression. **E** Percentage of viable ESCC cells with or without ZNF154 overexpression. Data are expressed as absorbance. **F** Number of colonies formed by ESCC cells with or without ZNF154 overexpression. **G** Migration rates of ESCC cells with or without ZNF154 overexpression. Left panel: representative image of the Transwell assay; right panel: quantitative analysis. Experiments were repeated for two times with the same trend and the data presented are mean ± SD of one independent experiments. *P < 0.05; **P < 0.01; ***P < 0.001. Statistical analysis: Student’s *t* test (two sided). OE overexpression of ZNF154. NC and vectors are both negative controls.

Based on the results above, we hypothesized that the hypermethylation of ZNF154 promoter likely plays an important role in the development of ESCC by downregulating the expression levels of ZNF154. In contrast to the promoter methylation levels, that of the ZNF154 mRNA was significantly lower in the ESCC tissues compared to the NATs (Fig. 2A). Furthermore, ESCC cell lines treated with 5-aza-2’ deoxycytidine (5-aza-dC), a potent inhibitor of DNA methyltransferase, showed a marked reduction in the methylation level of ZNF154 promoter with a concomitant upregulation of ZNF154 mRNA (Fig. 2B, C). This clearly indicated that hypermethylation of the ZNF154 promoter downregulates the transcript levels. Overexpression of ZNF154 in the ESCC cell lines significantly inhibited their proliferative and migration capacities compared to that of the controls (Fig. 2D–G), indicating that ZNF154 functions as a tumor suppressor gene in ESCC.

### Targeted demethylation of ZNF154 promoter inhibited the malignant potential of ESCC cells

**Fig. 3:**
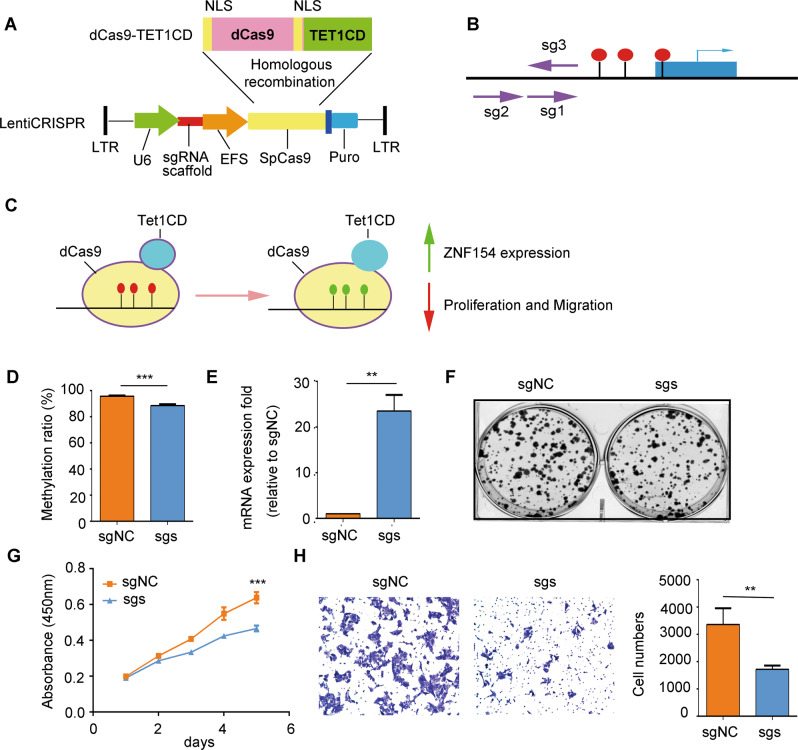
Targeted demethylation at ZNF154 promoter induces the expression of ZNF154 and inhibits the proliferation and migration of ESCC cells. **A** Schematic diagram of the CRISPR demethylation plasmid. **B** Diagram of the sgRNAs (shown as arrows) designed to target the hypermethylation region of ZNF154 promoter. Arrowhead indicates the 3ʹ end of the sgRNAs. **C** A CRISPR-dCas9 fusion protein was designed to target the ZNF154 promoter and remove the methyl-CpGs in ZNF154 promoter. The red and green circles respectively indicate the methylated and unmethylated CpGs. **D** MR of the ZNF154 promoter in Kyse30 cells expressing sgRNAs and sgNC with the dCas9-Tet1CD fusion protein**. E** ZNF154 mRNA levels in Kyse30 cells expressing sgRNAs and sgNC with the dCas9-TET1CD fusion protein. **F** No. of colonies formed by Kyse30 cells expressing sgRNAs and sgNC. **G** Viability of Kyse30 cells that express sgRNAs and sgNC. Data are expressed as absorbance. **H** Migration rate of Kyse30 cells expressing sgRNAs and sgNC. Left panel: representative image of the Transwell assay; right panel: quantitative analysis. Data presented are mean ± SD of three independent experiments. *P < 0.05; **P < 0.01; ***P < 0.001. Statistical analysis: Student’s *t* test (two sided). sg: sgRNA. sgs: sg1 + sg2 + sg3. sgNC sgRNA for negative control, NLS nuclear localization signal, U6 promoter, EFS promoter, SpCas9 S. pyogenes Cas9, Puro puromycin, LTR long terminal repeat.

The hypermethylated region of the ZNF154 promoter was targeted using the CRISPR tool. Briefly, the Cas9 sequence of the LentiCRISPR plasmid was replaced with dCas9-Tet1CD and three sgRNAs targeting the hypermethylation region of ZNF154 promoter were respectively inserted into the construct (Fig. 3A, B). The ESCC cells were infected with the lentiviruses, and the sgRNAs recruited the dCas9-Tet1CD fusion protein to this region, thereby allowing the Tet1 catalytic domain to induce DNA demethylation (Fig. 3C). We detected significant demethylation of the ZNF154 promoter in the CRISPR cells compared to the controls, along with upregulation of ZNF154 mRNA (Fig. 3D, E). Furthermore, demethylation of the ZNF154 promotor inhibited the proliferation and migration of ESCC cells (Fig. 3F–H). Taken together, hypermethylation of ZNF154 promoter increases the proliferation and migration of ESCC cells by repressing ZNF154 mRNA levels.

### Targeted demethylation of ZNF154 promoter inhibited the proliferation and migration of ESCC cells in vivo

**Fig. 4:**
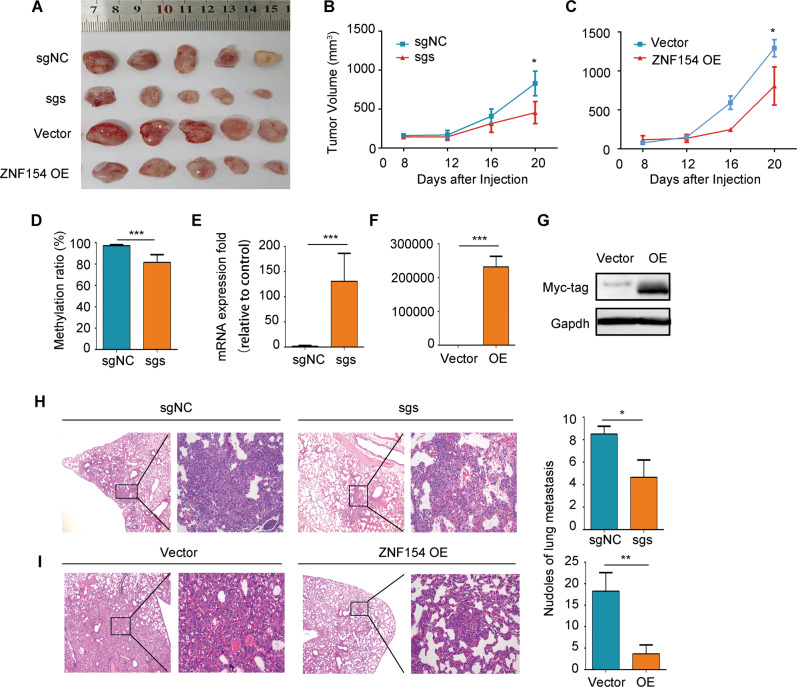
Targeted demethylation strategy in vivo. **A** Representative images of the tumors derived from Kyse-30 cells with targeted demethylation of ZNF154 promoter and ZNF154 overexpression. **B**, **C** Tumor growth curves of the indicated groups. **D** MR of the hypermethylated region at ZNF154 promoter following targeted demethylation. **E**, **F** ZNF154 mRNA levels in demethylation and overexpression groups. **G** The levels of Myc tag and GAPDH in the ZNF154 overexpression group. **H**, **I** Representative images of HE-stained lung metastatic nodules (magnification: left, 4×; right, 20×) and the number of metastatic nodules. Upper: Demethylation group (**H**); Lower: Overexpression group (**I**). Animal experiments were repeated for two times with the same trend and the data presented are mean ± SD of one independent experiments. *P < 0.05; **P < 0.01; ***P < 0.001. Statistical analysis: Student’s *t* test (two sided). Demethylation group: sgNC and sgs; Overexpression group: vector and ZNF154 OE. sg: sgRNA. sgs: sg1 + sg2 + sg3. sgNC sgRNA for negative control, OE overexpression of ZNF154.

In order to study the long-term effect of dCas9-Tet1CD-induced demethylation on the growth of ESCC cells in vivo, we established a subcutaneous xenograft model by inoculating Kyse-30 cells with targeted demethylation or ZNF154 overexpression in BALB/c mice. Compared to the control group, the tumors derived from the ESCC cells with ZNF154 overexpression or promoter demethylation were significantly smaller (*P* < 0.05) (Fig. 4A). The mean volume of the ZNF154 methylated and demethylated Kyse-30 xenografts were 830.1 ± 157.6 mm^3^ and 456.1 ± 141.5 mm^3^ respectively, and that of control and ZNF154-overexpressing Kyse-30 xenografts were 1293.0 ± 110.3 mm^3^ and 807.7 ± 244.6 mm^3^ respectively (Fig. 4B, C). The MR of the ZNF154 promotor was significantly lower in the demethylated versus control xenograft tissues (*P* < 0.001) (Fig. 4D). The effect of targeted demethylation was also verified by bisulfite sequencing (data is not shown). The ZNF154 mRNA levels were upregulated in the demethylated group (*P* < 0.001) (Fig. 4E), as well as in the ZNF154-overexpressing Kyse-30 xenografts (Fig. 4F, G). The kyse-30 cells with targeted demethylation or ZNF154 overexpression were also injected into the tail vein of BALB/c mice to establish a metastasis model. There were markedly fewer and smaller pulmonary metastatic nodules in the targeted demethylation (*P* < 0.05) and overexpression (*P* < 0.01) groups compared to the vector control group (Fig. 4H, I). Taken together, targeted demethylation of the ZNF154 promoter can induces its expression and inhibits the malignant progression of ESCC cells in vivo.

### ZNF154 acts as a transcription factor to upregulate the expression of ESCC-associated tumor suppressor genes

**Fig. 5:**
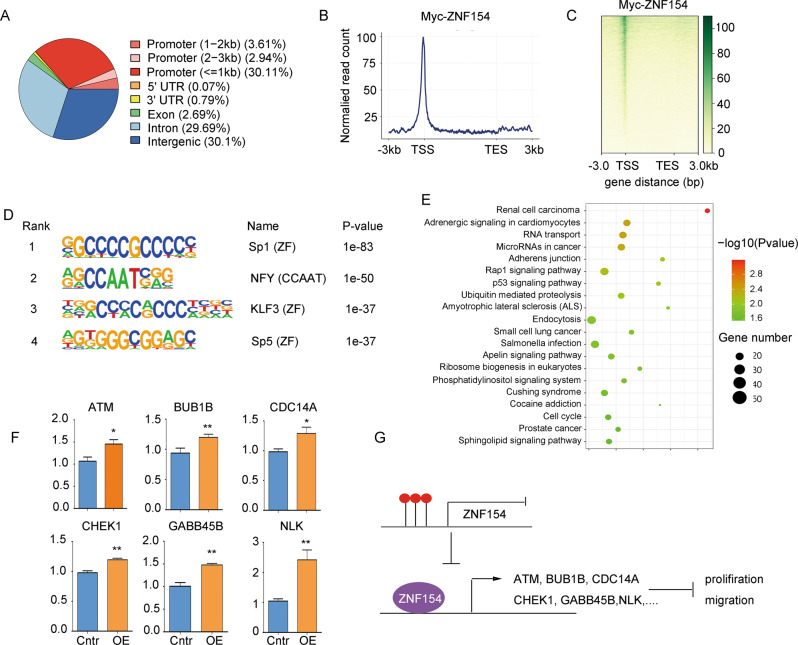
ZNF154 acts as a transcription factor to upregulate the expression of ESCC-associated tumor suppressor genes. **A** The genomic distribution of Myc-tag CUT&Tag peaked in kyse30 cells stably expressing Myc-tagged ZNF154. **B** The distribution of mapped ZNF154 peak locations waswithin 3 kb upstream and downstream of transcriptional start sites (TSSs) at genes with ZNF154-bound promoters. **C** CUT&Tag density heat map of ZNF154 enrichment in kyse30 cells stably expressing psin-Myc-ZNF154, within 3 kb around TSS. Gene order was arranged from highest to lowest density. **D** The top-enriched motifs at the ZNF154-binding sites in kyse30 cells stably expressing psin-Myc-ZNF154. *P* values were calculated based on hypergeometric distributions. **E** Enrichment KEGG pathway for differentially abundant genes bound by ZNF154 in kyse30 cells stably expressing psin-Myc-ZNF154. **F** mRNA levels of the indicated genes. **G** Regulation pattern of ZNF154 promoter hypermethylation promoting the proliferation and migration of ESCC cells. *P < 0.05; **P < 0.01. ZF zinc finger, Cntr control, OE overexpression of ZNF154.

To better understand the molecular mechanisms of ZNF154-inhibited proliferation and migration, we performed high-throughput CUT&Tag analysis. Due to lack of ChIP-grade ZNF154 antibody, we generated a kyse30 cell line expressing Myc-ZNF154. Subsequently, ChIP was also performed using anti-Myc antibodies. A total of 6702 annotated genome-binding peaks were identified, and a large number of peaks (~36.7%) were located in the promoter region of genes, 0–3 kb around the transcriptional start site (TSS), with such peaks exhibiting a high density around the TSS (Fig. 5A–C). De novo motif search analysis of ZNF154-binding sites revealed that ZNF154 was significantly enriched at zinc finger protein family binding sites, especially Sp1, KLF3 and Sp5, suggesting that ZNF154 may interact with these transcription factors to jointly regulate the transcription of downstream target genes (Fig. 5D). Then we performed KEGG pathway enrichment analysis on the target genes, and found that these genes were involved in cell cycle, adhesion junction and p53 signaling, playing an important role in regulating the proliferation and migration of ESCC cells (Fig. 5E). We used the GEPIA software to analyze the correlation between the expression of these genes and ZNF154 in esophageal cancer, and found that 12 genes correlated with the expression of ZNF154 (Supplementary Fig. 2). We detected the mRNA levels of these 12 genes in ZNF154 overexpressed cells by qPCR and found that the mRNA levels of six TSGs were higher than the negative control (Fig. 5F). The six TSGs were involved in regulating the pathways of cell cycle, p53 signaling and Wnt/β-catenin signaling, which played an important role in the proliferation and migration of ESCC cells [26**–**32]. Based on the above results, it could be inferred that ZNF154 inhibits the proliferation and migration of ESCC cells by transcriptionally upregulating the expression of ESCC-related tumor suppressor genes (Fig. 5G).

## Discussion

DNA methylation is an epigenetic regulatory mechanism that controls the expression of genes involved in cancer initiation and progression. Several genes have been identified that are aberrantly methylated in tumors, and are therefore potential diagnostic and prognostic markers. Furthermore, given that DNA methylation is reversible, it is also a promising therapeutic target for cancer [33, 34]. For instance, DNA methyltransferase inhibitors (DNMTis) such as 5­azacytidine (azacytidine) and 5­aza­2’­deoxycytidine (decitabine) can reverse silencing of tumor suppressor genes (TSGs) by blocking DNA methyltransferases (DNMTs) [35]. DNMTis are currently used for treating hematological neoplasms [36], but are not effective against solid tumors due to their non-specificity [37].

No study so far has reported on the therapeutic efficacy of epigenetically editing the genes involved in esophageal cancer [38**–**40]. Hu et al developed a CRISPR-based approach for targeted DNA demethylation in HEK-293FT, SH-SY5Y and HeLa cell lines [12]. However, the demethylation effect was transient and could not achieve long-term results in vivo. The LentiCRISPR plasmid, which is adapted from Zhang et al, can constitutively expresses the sgRNA along with Cas9 protein to achieve stable gene knockout. In addition, the purinomycin gene in this plasmid allowed rapid selection of the stably infected cells [41]. We established a targeted demethylation strategy based on the LentiCRISPR technology and the dCas9/MS2 system [12]. Since the dCas9/MS2 system cannot be used to package the lentivirus for a constant demethylation effect, we replaced Cas9 with dCas9-Tet1CD. Therefore, the LentiCRISPR/dCas9-Tet1CD plasmid can constitutively express the sgRNA with the dCas9-Tet1CD fusion protein for stable gene demethylation.

Although several TSGs are hypermethylated in ESCC, most have low diagnostic efficiency or lack the robust validation studies to merit clinical application [33]. We identified ZNF154 as a TSG in ESCC, and that its promoter is hypermethylated in ESCC tissues and EECs of the patients. In addition, the hypermethylated ZNF154 promoter showed high diagnostic efficiency for ESCC with 89.33% sensitivity and 93.65% specificity. The hypermethylation of ZNF154 promoter was also correlated with poor prognosis, indicating its prognostic relevance as well. Consistent with our results of 5-aza-dC, our targeted demethylation strategy decreased the methylation ratio of ZNF154 promoter by 8% in vitro and 15% in vivo. In addition, the ZNF154 mRNA level was upregulated by 15 to 20-fold in vitro and 100 to 200-fold in vivo. Thus, our targeted demethylation strategy achieved long-term effect in vivo. However, we were not able to confirm the changes in ZNF154 protein level due to the lack of specific antibodies. Nevertheless, targeted demethylation inhibited the malignant potential of ESCC cells in vivo. Therefore, epigenetic editing is a promising targeted therapeutic strategy for ESCC with aberrant DNA methylation.

To further explore the mechanism of the inhibitory effect of ZNF154, we conducted the high-throughput sequencing analysis for the downstream target genes of ZNF154 using CUT&Tag, and found that ZNF154 acts on the promoter of the target genes to transcriptionally regulate gene expression. We found the expression of 6 TSGs promoted by ZNF154 through the prediction of GEPIA software and the verification of qPCR. These tumor suppressor genes were involved in regulating the pathways of cell cycle, p53 signaling and Wnt/β-catenin signaling, which played an important role in the proliferation and migration of ESCC cells [26**–**32]. However, the specific mechanism remains to be further explored in future. Studies have reported that sulforaphene might have a potential in inhibiting the progression of ESCC by activating the GADD45B-MAP2K3-P38-p53 signaling pathway [31], indicating the possibility to improve the therapeutic effect of ESCC patients with aberrant ZNF154 methylation through epigenetic editing combined with targeted drugs like sulforaphene.

## Materials and methods

### Biological samples

Based on bisulfite sequencing, the CpG methylation statuses were evaluated in nine ESCC tissues, four unpaired normal adjacent tissues (NATs), as well as two ESCC cell lines. The methylation ratios (MRs) were evaluated in cohort I (78 ESCC tissues, 21 NATs and 16 healthy human leucocytes) and cohort II (189 normal people and 75 ESCC patients) by ddPCR, respectively. Tumor specimens from cohort I (78 ESCC tissues) were used to analyze the correlation between clinical outcome and the MRs of ZNF154 promoter. The EECs from cohort II were used to analyze the diagnostic efficiency of the MRs of ZNF154 promoter.

All of the patients were pathologically diagnosed and confirmed with ESCC. The ESCC and normal adjacent tissues, and healthy human leukocytes were obtained from Sun Yat-Sen University Cancer Center (SYSUCC). The esophageal exfoliated cells (EECs) of the ESCC patients and healthy individuals (including those with difficulty in swallowing, esophagitis, gastritis and Barrett’s esophagus) were retrieved from SYSUCC, Anhui Provincial Cancer Hospital (Hefei, China) and Shantou University Cancer Hospital respectively. The age, sex, tumor location, TNM stage (according to the AJCC/UICC Cancer Staging Manual, 8th ed.), pathological type and pathological grade data were collected for all subjects (Supplementary Tables 1 and 2). The patients had been followed-up till Nov 2020 for survival analysis. The study was approved by the Research Ethics Committee of SYSUCC (reference number: GZR2020–277) and written informed consent was obtained from all patients for the use of their data.

### DNA isolation and bisulfite conversion

Total DNA from paraffin-embedded tissues, cell lines and EECs were isolated using specific kits (Targene Medical, Wuhu, China) according to the manufacturer’s instructions. Zymo EZ DNA Methylation Kit (Zymo Research, Orange County, California, USA) was used for bisulfite conversion.

### Bisulfite sequencing

The bisulfite-treated DNA was amplified using specific primers by PCR. The target fragments were purified using a kit (TIANGEN, Beijing, China) and sequenced by cloning (Vazyme, Nanjing, China).

### Methylation analysis by droplet digital PCR (ddPCR)

The bisulfite-converted DNA template (5–10 ng/µl) was used for methylation-specific PCR using the 2× ddPCR Supermix, including ddPCR Master MIX, primers and probes of methylated and unmethylated ZNF154 gene (Donated by Targene Medical, Wuhu, China) and QX200™ Droplet Digital™ PCR System (Bio-Rad, Hercules, California, USA). The methylation ratio of ZNF154 promoter was calculated using the following equation: methylated droplets (MD)/(MD + unmethylated droplets (UMD). The primer sequences can be requested from the author.

### Acquisition of EECs

The EECs of healthy individuals were collected using the cytosponge capsule. Briefly, the subjects were instructed to swallow the cytosponge capsule, which was transported to the stomach by peristalsis and subsequently dissolved. The contents of the capsule expanded into a spherical sponge, which was then pulled up along the esophagus along with the adsorbed EECs [42]. The EECs of ESCC patients were obtained by scraping the surface of resected tumors with a cytobrush.

### Cell culture and 5-aza-20-deoxycytidine treatment

Human esophageal squamous carcinoma cell lines (Kyse-30 and Kyse-140) were purchased from CellCook Company (Guangzhou, China), and the 293 T cell line was kindly provided by Dr. Yuan-Zhong Wu (Department of Experimental Research, Sun Yat-sen University). All cell lines were authenticated on the basis of viability, recovery, growth, morphology and isoenzymology by the provider. The ESCC cells were cultured in RPMI 1640 medium (ThermoFisher, Waltham, Massachusetts, USA) and the 293 T cell line in DMEM (ThermoFisher), each supplemented with 10% fetal bovine serum (FBS; ThermoFisher), 100 U/ml Penicillin and 100 µg/ml Streptomycin (ThermoFisher). The cells were incubated at 37 °C under 5% (vol/vol) CO_2_, and media was changed daily. Cultures were passaged at the ratio of 1:3 every two days using 0.25% trypsin (ThermoFisher). For 5-aza-20-deoxycytidine (5-aza-dC; Sigma-Aldrich, Darmstadt, Germany) treatment, ESCC cell lines were seeded in 6-well plates at the density of 2 × 10^5^ cells per well, and cultured with 2 µM 5-aza-dC for four days. The medium was replaced once after 48 h.

### Construction of ZNF154-overexpressing ESCC cell lines

ZNF154 cDNA from healthy human leukocytes was inserted into a lentiviral backbone containing a puromycin selection cassette and three tag proteins Myc. The amplified ZNF154 cDNA was cloned into the psin vector, which was kindly provided by Dr. Yuan-Zhong Wu (Department of Experimental Research, Sun Yat-sen University). Briefly, the psin vector was digested with BstBI (NEB, Beijing, China) and the ZNF154 fragment was inserted via homologous recombination (Vazyme). The ESCC cells were transduced with the lentivirus, and the stably infected cells were selected by three days of puromycin selection (1 µg/mL). The primers used for cloning are listed in Supplementary Table 3.

### Quantitative real-time PCR analysis

Total RNA were isolated from tissues and cell lines using Trizol reagent according to the manufacturer’s instructions, and 1 μg RNA was reverse transcribed using a cDNA synthesis kit (TIANGEN). Quantitative real-time PCR (qRT-PCR) was performed using the SYBR Green Master Mix (Vazyme) on the AB7500 instrument (DELL, Texas, USA). Each sample was analyzed in triplicate, and normalized to GAPDH. Data were analyzed using the 2^−ΔΔCT^ calculation method. The primers are listed in Supplementary Table 4.

### Western blotting

Western blotting was performed as per the standard protocol using the anti-MYC tag antibody (at 1:1000 dilution; Cat.no: ab62928, Abcam plc, Cambridge, UK), with GAPDH (at 1:5000 dilution; Cat.no: ab8245, Abcam plc, Cambridge, UK) as the loading control. The protein bands were detected using Super ECL Detection Reagent (YEASEN, Shanghai, China).

### In vitro functional assays

For the colony formation assay, 1 × 10^3^ cells were seeded in 6-well culture plates. After nine days of culture, the ensuing colonies were fixed with methyl alcohol, stained with crystal violet, and counted. To evaluate the proliferation rate, 1 × 10^3^ cells were seeded per well into 96-well plates, and cultured for five days. Ten microliters CCK8 reagent (DOJINDO, Shanghai, China) was added to each well at the stipulated time points, and the absorbance values were measured at 450 nm. The migration capacity of the cells was tested with the transwell assay. Briefly, the cells were seeded into the top chamber of each transwell insert in a 24-well plate at the density of 6 × 10^5^ cells/ml in 200 µl serum-free media, and each lower chamber was filled with 800 µl RPMI 1640 medium supplemented with 20% FBS. The cells were cultured for 24 h, and the number of migrated cells were counted under a microscope (NIKON, Tokyo, Japan).

### Lentiviral CRISPR/dCas9-Tet1CD plasmid cloning

The dCas9-Tet1 catalytic domain was cloned from the pdCas9-Tet1-CD plasmid (Addgene, plasmid #83340). The backbone of LentiCRISPR plasmid without Cas9 was cloned and ligated with dCas9-Tet1CD through homologous recombination (Vazyme) to generate the lentiviral CRISPR/dCas9-Tet1CD plasmid. This lentiviral backbone contains purinomycin for screening infected cells. The LentiCRISPR plasmid was kindly provided by Dr. Yuan-Zhong Wu (Department of Experimental Research, Sun Yat-sen University). The complete sequence of LentiCRISPR plasmid and additional information can be found at the website: www.genome-engineering.org. The primers used for cloning are listed in Supplementary Table 5.

### Transfection of ESCC cell lines

CRISPR sgRNA1, 2 and 3 sequences and a non-specific control sgRNA were respectively inserted into the lentiviral CRISPR/dCas9-Tet1CD plasmid. The ESCC cells infected twice with the lentivirus, and selected using purinomycin (1 µg/mL). The stably transduced cells were cultured for 15–20 days prior to methylation analysis and RNA expression analysis. The primer sequences can be requested from the author.

### In vivo assay

Kyse30 cells expressing ZNF154 protein and sgRNAs with dCas9-Tet1CD fusion protein were injected subcutaneously (4 × 10^6^ cells in 0.2 ml phosphate buffer) into the right thoracic side of 7-weeks-old male Balb/c nude mice (*n* = 5). Tumor volume (V, mm^3^) was measured every four days for 20 days starting eight days after implantation using the following formula: V = L × W^2^/2 (L – largest diameter in mm, and W – smallest diameter in mm). For the metastatic model, the Kyse30 cells (1 × 10^6^ cells in 0.15 ml phosphate buffer) were injected into the tail vein of male Balb/c nude mice. After eight weeks, the lungs were dissected, fixed with 10% neutral formalin and embedded with paraffin for hematoxylin and eosin (HE) staining. The tumor tissues were processed for molecular analyses (methylation assay, RT-PCR and western blotting) as already described. Experiments involving animals were used randomly and approved by the Animal Ethics Committee of SYSUCC.

### High-throughput CUT&Tag

CUT&Tag analysis was performed as described in a previous study [43]. The primary antibody was the HRP Anti-Myc tag antibody (at 1:50 dilution; Abcam plc, ab62928, Cambridge, UK), with more details provided in the Supplementary Information 1. The raw sequencing data can be requested from the authors.

### Statistical analysis

Data was analyzed using SPSS22.0 software (SPSS, Chicago, Illinois, USA). X^2^ test or fisher’s exact test was used to analyze the association between ZNF154 promoter methylation and clinicopathological parameters. Survival curves were plotted by the Kaplan–Meier method and compared using the log-rank test. Cytological experiments were repeated for two or three times. All data were expressed as mean ± standard deviation (SD). T test was used to compare any two groups, and one-way ANOVA was used for multiple comparisons. Two-sided *P* < 0.05 was considered statistically significant.

## Supplementary information

supplementary materials

## Data Availability

All data included in this study are available upon request by contact with the corresponding author.
