# Supplementary material for: Targeted demethylation at ZNF154 promotor upregulates ZNF154 expression and inhibits the proliferation and migration of Esophageal Squamous Carcinoma cells
Source: Oncogene. 2022 Sep 5;41(40):4537–46. doi: 10.1038/s41388-022-02366-y (PMC9525237; doi:10.1038/s41388-022-02366-y)
Supplement: Supplementary file 1 — supplementary materials [file 41388_2022_2366_MOESM1_ESM.docx]

**Supplementary Figure 1**


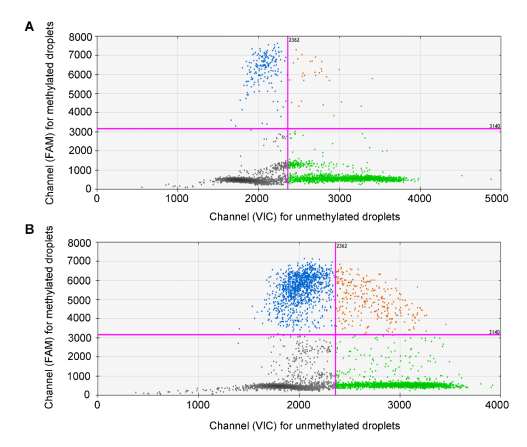


**Supplementary Figure 1.** The methylation ratio of ZNF154 promoter was detected by ddPCR. The scatter plots showing the intensity of methylated droplets (FAM channel) and unmethylated droplets (VIC channel) detected by ddPCR in NATs **(A)** and ESCC tissues **(B)**. Manual gating (pink line) highlights the methylated droplets (blue dots) and unmethylated droplets (green dots).

**Supplementary Figure 2**


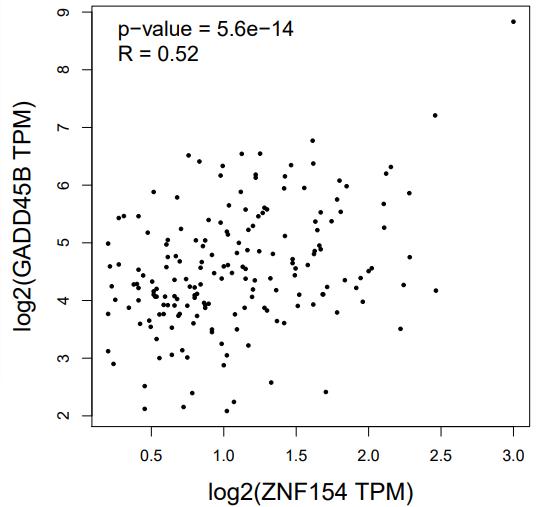

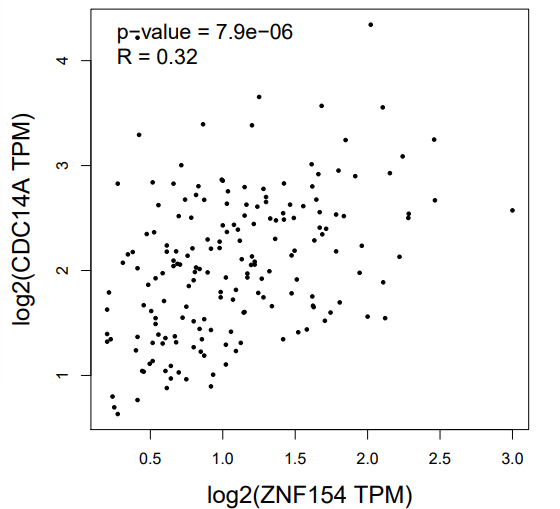

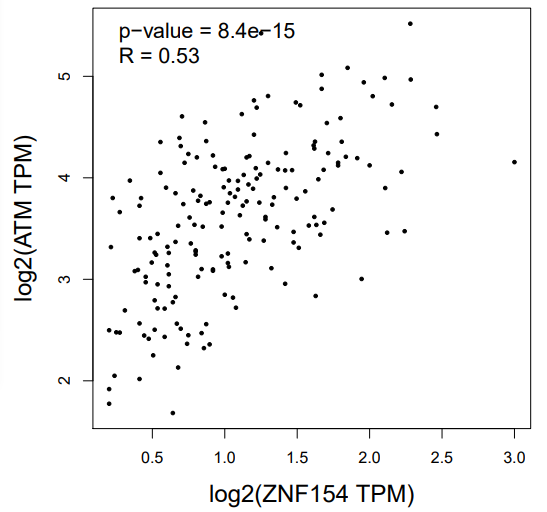


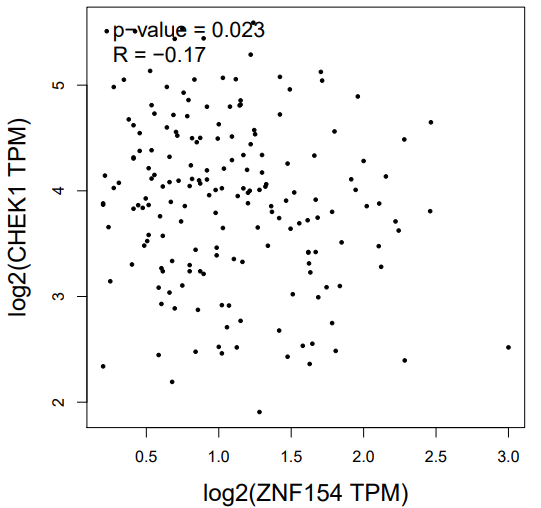

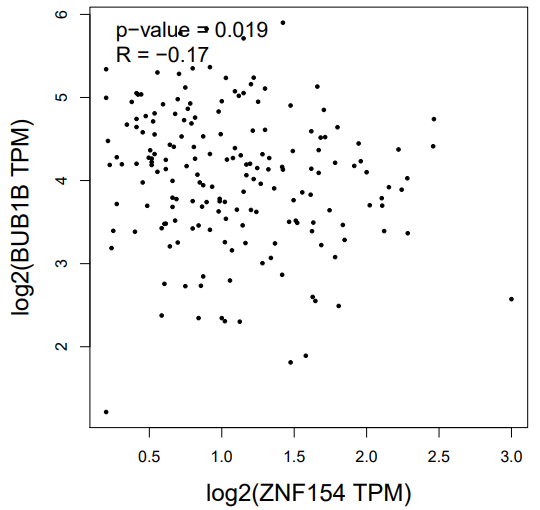

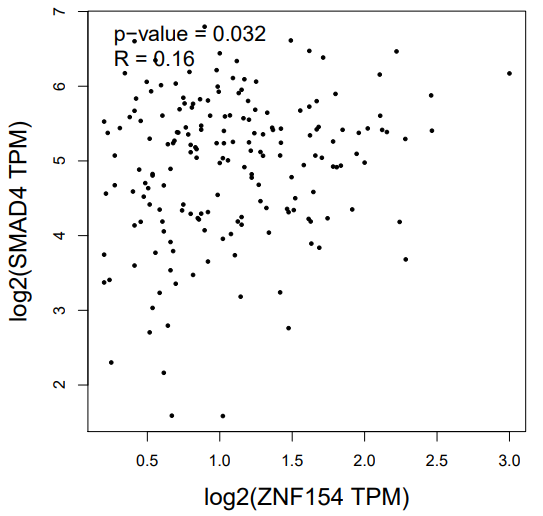


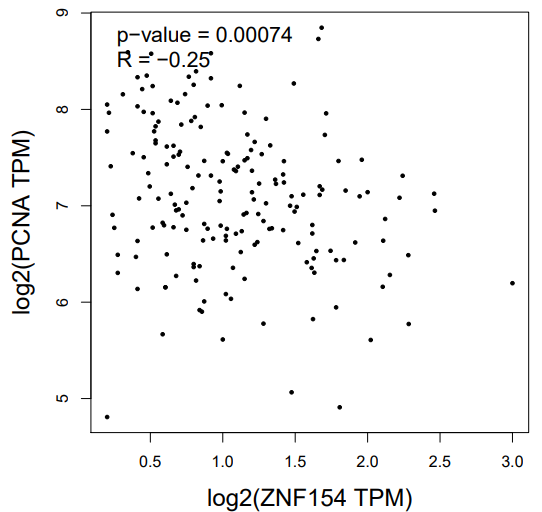

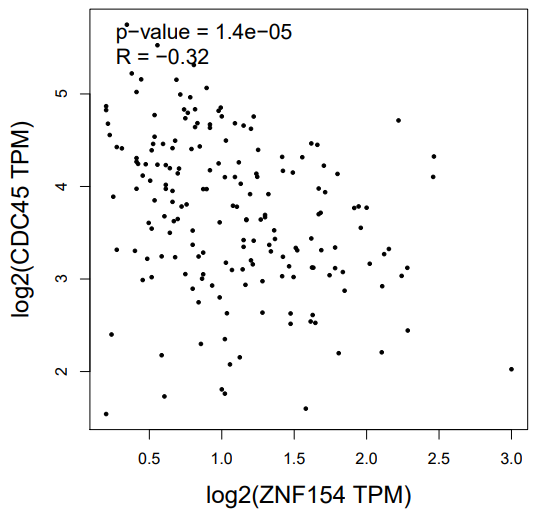

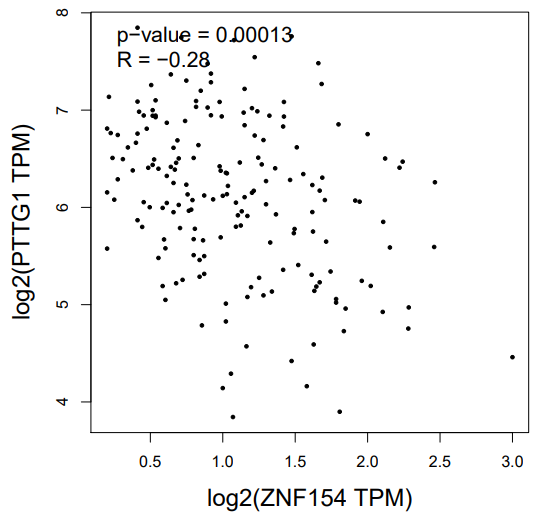


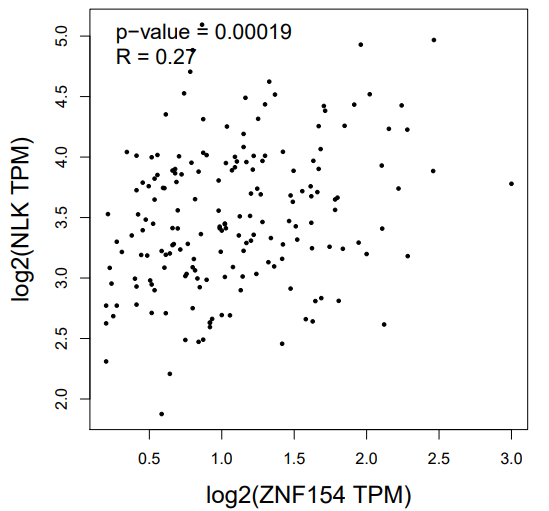

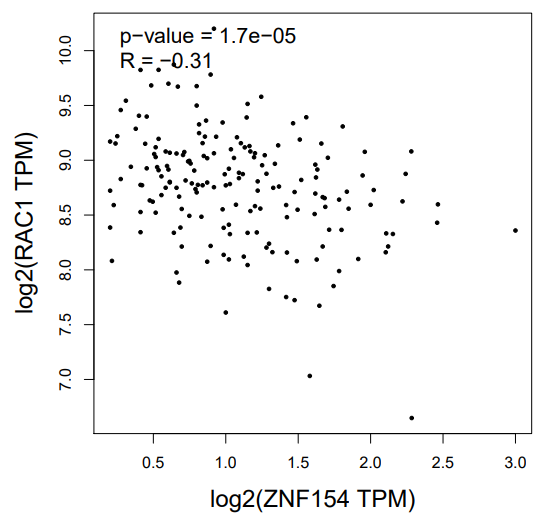

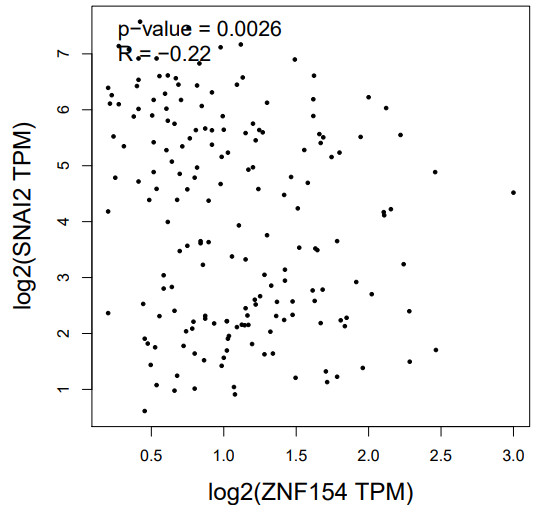


**Supplementary Figure 2.** The correlation between the expression of ZNF154 and the 12 genes from KEGG was analyzed by the GEPIA software. The higher the R value, the higher the correlation. If the R < 0, it means the negative correlation. If the R > 0, it means the positive correlation. Two-sided P < 0.05 was considered statistically significant.

**Supplementary Table 1. The demographic and clinico-pathological characteristics of ESCC tissues, NATs and white blood cells**

| **Characteristic** | **ESCCs**  (N=78, %) | **NATs**  (N=19, %) | **White cells**  (N=16, %) | ***P value*** |
| --- | --- | --- | --- | --- |
| Sex |  |  |  | 0.169* |
| Female | 13 (16.7) | 4 (19.0) | 6 (37.5) |  |
| Male | 65 (83.3) | 15 (71.4) | 10 (62.5) |  |
| Age, years old |  |  |  | < 0.001^#^ |
| Mean | 62 | 64 | 47 |  |
| Range | 42-88 | 51-78 | 29-59 |  |
| Pathologic grade |  |  |  | 0.609* |
| I | 28 (35.9) | 8 (42.1) | NA |  |
| II-III | 50 (64.1) | 11 (57.9) | NA |  |
| TNM stage |  |  |  | 1.000* |
| I-II | 38 (48.7) | 9 (47.4) | NA |  |
| III-IV | 40 (51.3) | 10 (52.6) | NA |  |
| T stage |  |  |  | 0.387* |
| T1-2 | 21 (26.9) | 3 (15.8) | NA |  |
| T3-4 | 57 (73.1) | 16 (84.2) | NA |  |
| N stage |  |  |  | 0.609* |
| N0 | 34 (43.6) | 10 (52.6) | NA |  |
| N1-3 | 44 (56.4) | 9 (47.4) | NA |  |
| Tumor location |  |  |  | 0.596* |
| Upper/Middle | 54 (69.2) | 12 (63.2) | NA |  |
| Lower | 24 (30.8) | 7 (36.8) | NA |  |

P values (*) were calculated using χ2 test, or Fisher’s exact test.

P value (#) was calculated using one-way ANOVA test.

Abbreviations: TNM, tumor-node-metastasis. NAT, unpaired normal adjacent tissues. ESCC, esophageal squamous carcinoma. N, number of cases. NA, not applicable.

**Supplementary Table 2. The demographic and clinico-pathological characteristics of EECs**

| **Characteristic** | **ESCC**  **patients** | **Normal people** | ***P value*** |
| --- | --- | --- | --- |
| Total (N) | 75, % | 189, % |  |
| Sex |  |  | < 0.001* |
| Female | 15 (20.0) | 99 (52.5) |  |
| Male | 60 (80.0) | 90 (47.5) |  |
| Age, years old |  |  | < 0.001^#^ |
| Mean | 62 | 49 |  |
| Range | 43-79 | 22-73 |  |
| TNM stage |  |  |  |
| I | 2 (2.7) | NA |  |
| II | 17 (22.7) | NA |  |
| III | 25 (33.3) | NA |  |
| IV | 26 (34.7) | NA |  |
| NA | 5 (6.7) |  |  |
| Pathologic grade |  |  |  |
| I | 1 (1.3) | NA |  |
| II | 36 (48.0) | NA |  |
| III | 18 (24.0) | NA |  |
| NA | 20 (26.7) |  |  |
| Tumor site |  |  |  |
| Upper | 11 (14.7) | NA |  |
| Middle | 39 (52.0) | NA |  |
| Lower | 18(24.0) | NA |  |
| NA | 7 (9.3) |  |  |

P values (*) were calculated using χ2 test, or Fisher’s exact test.

P value (#) was calculated using t test.

Abbreviations: TNM, tumor-node-metastasis. ESCC, esophageal squamous carcinoma. N, number of cases. NA, not applicable.

**Supplementary Table 3. Primers used for cloning psin-ZNF154 plasmid**

| **Primers** | **Sequence** |
| --- | --- |
| Forward primer for ZNF154 | agacttgaccggtagtttcgaaatggcagcggccactctg |
| Reverse primer for ZNF154 | gatcactagtgctagcttcgaattatcgactatgaattctctgatgtttaataaggc |

**Supplementary Table 4. qPCR primers**

| **Primers** | **Sequence** |
| --- | --- |
| Forward primer for ZNF154 | TGTGGCCGTACACTTCTCCT |
| Reverse primer for ZNF154 | TGCAGGTCTTCACAAACAAGG |
| Forward primer for GAPDH | TGACTTCAACAGCGACACCC |
| Reverse primer for GAPDH | CTGGTGGTCCAGGGGTCTTA |
| Forward primer for ATM | ATCTGCTGCCGTCAACTAGAA |
| Reverse primer for ATM | GATCTCGAATCAGGCGCTTAAA |
| Forward primer for BUB1B | AAATGACCCTCTGGATGTTTGG |
| Reverse primer for BUB1B | GCATAAACGCCCTAATTTAAGCC |
| Forward primer for CDC14A | GGGGAACTAATCGGGGCTTG |
| Reverse primer for CDC14A | CAGCGGTCCAAAATCTGCATA |
| Forward primer for CHEK1 | ATATGAAGCGTGCCGTAGACT |
| Reverse primer for CHEK1 | TGCCTATGTCTGGCTCTATTCTG |
| Forward primer for GADD45B | TACGAGTCGGCCAAGTTGATG |
| Reverse primer for GADD45B | GGATGAGCGTGAAGTGGATTT |
| Forward primer for NLK | CGCAAAAATGATGGCGGCTTA |
| Reverse primer for NLK | CCCAGGGTTTAACATGGCTG |

**Supplementary Table 5. Primers used for cloning lentiviral CRISPR/dCas9-Tet1CD plasmid**

| **Primers** | **Sequence** |
| --- | --- |
| Forward primer for dCas9-Tet1CD | cgctgccaccatgtacccatacgatgttccagattacgcttcg |
| Reverse primer for dCas9-Tet1CD | agtttgttgcgacccaacgattgtagggtcccgc |
| Forward primer for LentiCRISPR without Cas9 | tcgttgggtcgcaacaaacttctctctgctgaaacaagccg |
| Reverse primer for LentiCRISPR without Cas9 | atgggtacatggtggcagcgctctagaaccggtc |

**Supplementary information 1.**

**High-throughput CUT&Tag**

CUT&Tag assay was performed as described previously with modifications (Hatice et al., 2019 ). Briefly, 100000 cells were washed twice gently with wash buffer (20mM HEPES pH 7.5; 150mM NaCl; 0.5mM Spermidine; 1× Protease inhibitor cocktail). 10μL Concanavalin A coated magnetic beads (Bangs Laboratories) were added per sample and incubated at RT for 10min. Remove unbound supernatant and resuspended beadbound cells with dig wash buffer (20mM HEPES pH 7.5; 150mM NaCl; 0.5mM Spermidine; 1× Protease inhibitor cocktail; 0.05% Digitonin; 2mM EDTA) and a 1:50 dilution of primary antibody or IgG control antibody (normal rabbit IgG：Millipore cat.no. 12-370，normal mouse IgG：Millipore cat.no. 12-371) incubated on a roating platform overnight at 4℃. The primary antibody was removed using magnet stand. Sencondary antibody (Rabbit Anti-Mouse IgG H&L：abcam，ab611709 ；Anti-Rabbit IgG antibody, Goat monoclonal: Millipore AP132) was diluted 1:100 in dig wash buffer and cells were incubated at RT for 60 min. cells were washed using the magnet stand 2-3 times in dig wash buffer. A 1:100 dilution of pA-Tn5 adapter complex was prepared in dig-med buffer (0.01% Digitonin; 20mM HEPES pH 7.5; 300mM NaCl; 0.5mM Spermidine; 1× Protease inhibitor cocktail) and incubated with cells at RT for 1h. Cells were washed 2–3× for 5 min in 1 mL Dig-med buffer. Then cells were resuspended in tagmentation buffer (10mM MgCl2 in Dig-med Buffer) and incubated at 37°C for 1h. DNA was purified using phenol-chloroform-isoamyl alcohol extraction and ethanol precipitation. To amplify libraries, 21μL DNA was mixed with 2μL of a universal i5 and auniquely barcoded i7 primer. A volume of 25 μL NEBNext HiFi 2× PCR Master mix was added and mixed. The sample was placed in a Thermocycler with a heated lid using the following cycling conditions: 72°C for 5 min (gap filling); 98°C for 30s; 14 cycles of 98°C for 10s and 63°C for 30s; final extension at 72°C for 1 min and hold at 8°C. libraried clean-up was performed XP beads (Beckman Counter).

**DNA sequencing**

The size distribution of libraries was determined by Agilent 4200 TapeStation analysis, and libraries were mixed to achieve equal representation as desired aiming for a final concentration as recommended by the manufacturer. Sequencing was performed in the Illumina Novaseq 6000 using 150bp paired-end following the manufacturer’s instructions.

**Data Analysis**

1. **Quality control**

Raw data (raw reads) of fastq format were firstly processed through in-house perl scripts. In this step, clean data (clean reads) were obtained by removing reads containing adapter, reads containing ploy-N and low quality reads from raw data. At the same time, Q20, Q30 and GC content the clean data were calculated. All the downstream analyses were based on the clean data with high quality.

1. **Reads mapping to the reference genome**

Mapping of pair-end reads. Before read mapping, clean reads were obtained from the raw reads by removing the adaptor sequences. The clean reads were then aligned to reference genome sequences using the bwa program.

1. **Call peak**

The bam file generated by the unique mapped reads as an input file, using macs2 software for callpeak with cutoff q value < 0.05.

1. **Motif analysis**

The HOMER's findMotifsGenome.pl tool was used for Motif analysis. The input file is the peak file and the genome fasta file. The DNA sequence is extracted according to the peak file, and the sequence is compared with the Motif database to obtain the Motif.

1. **Peak annotation**

Peaks were annotated by using homer's annotatePeaks.pl.

1. **Peak analysis**

Count the results of the annotations and plot the distribution results using R.

1. **GO and KEGG enrichment analysis**

GO Analysis: Gene ontology (GO) analysis was performed to facilitate elucidating the biological implications of unique genes in the significant or representative profiles of the gene in the experiment [Ashburner M, et al. Gene ontology: tool for the unification of biology. The Gene Ontology Consortium. Nat Genet. 2000 May;25(1):25-9.]. We downloaded the GO annotations from NCBI (http://www.ncbi.nlm.nih.gov/), UniProt (http://www.uniprot.org/) and the Gene Ontology (http://www.geneontology.org/). Fisher’s exact test was applied to identify the significant GO categories and FDR was used to correct the p-values.

Pathway analysis was used to find out the significant pathway of the genes according to KEGG database. We turn to the Fisher’s exact test to select the significant pathway, and the threshold of significance was defined by P-value and FDR. [ Draghici S, et al. A systems biology approach for pathway level analysis. Genome Res. 2007 Oct;17(10):1537-45. Epub 2007 Sep 4.].

1. **Differential peak analysis**

We analysis differential accessible peak through 3 steps. First merge the peak files of each sample using the bedtools software. Second, The counts of the reads over the bed was determined for each sample using bedtools multicov. Finally, differential accessible peak was assessed using DESeq2. Region were called differentially accessible if the absolute value of the log2 fold change was 1 at an p value < 0.05.
